# Supplementary material for: Polyketide synthase gene clusters in the ericoid mycorrhizal fungus Oidiodendron maius: genome-wide prediction, expression profiling, and functional characterization of knockout mutants
Source: Front Plant Sci. 2026 Sep 16;17:1924430. doi: 10.3389/fpls.2026.1924430 (PMC13623903; doi:10.3389/fpls.2026.1924430)
Supplement: Supplementary file 1 [file SupplementaryFile1.zip › Supplementary Materials/Supplementary Folder 1.zip!/Oidma1_AssemblyScaffolds_Repeatmasked/knownclusterblast/cluster10/jgi.p_Oidma1_100660_mibig_hits.html]

| MIBiG Protein | Description | MIBiG Cluster | MiBiG Product | % ID | % Coverage | BLAST Score | E-value |
| --- | --- | --- | --- | --- | --- | --- | --- |
| AAT65717.1 | geranylgeranyl\_diphosphate\_synthase | BGC0000629 | Aflatrem\_biosynthetic\_gene\_cluster | 60 | 85.0267379679 | 387 | 1.6e-107 |
| BAD29970.1 | geranylgeranyldiphosphate\_synthase | BGC0000676 | Aphidicolin\_biosynthetic\_gene\_cluster | 58 | 78.3422459893 | 367 | 2.2e-101 |
| ctg1\_orf003 |  | BGC0000688 | Copalyl\_diphosphate\_biosynthetic\_gene\_cluster | 55 | 79.9465240642 | 354 | 1.5e-97 |
| AAW88510.1 | geranylgeranyl\_diphosphate\_synthase | BGC0001081 | Lolitrem\_biosynthetic\_gene\_cluster | 57 | 77.5401069519 | 347 | 1.8e-95 |
| AAK11531.1 | PaxG | BGC0001082 | Paxilline\_biosynthetic\_gene\_cluster | 54 | 77.0053475936 | 329 | 6.7e-90 |
| CBF85177.1 | conserved\_hypothetical\_protein | BGC0000673 | Ent-pimara-8(14),15-diene\_biosynthetic\_gene\_cluster | 46 | 84.2245989305 | 292 | 9.1e-79 |
| CAA75568.1 | geranylgeranyl\_diphosphate\_synthase | BGC0001270\_c3 | Gibberellin\_A3\_/\_gibberellin\_A1\_/\_gibberellin\_A7\_/\_gibberellin\_A4\_/ | 39 | 74.3315508021 | 210 | 2.7e-54 |
| BAV32147.1 | geranylgeranyl\_diphosphate\_synthase | BGC0001373 | Sordarin\_biosynthetic\_gene\_cluster | 35 | 105.080213904 | 200 | 2.8e-51 |
